# IRF1 is a core transcriptional regulatory circuitry member promoting AML progression by regulating lipid metabolism

**DOI:** 10.1186/s40164-025-00612-z

**Published:** 2025-03-01

**Authors:** Fenli Zhang, Zhiheng Li, Fang Fang, Yixin Hu, Zhixu He, Yanfang Tao, Yizhen Li, Zimu Zhang, Bi Zhou, Ying Yang, Yumeng Wu, Yijun Wu, Zhongling Wei, Ailian Guo, Ling Xu, Yongping Zhang, Xiaolu Li, Yan Li, Chunxia Yang, Man Zhou, Jian Pan, Shaoyan Hu, Xiaoyan Yang

**Affiliations:** 1 Department of Pediatrics, Affiliated Hospital of Guizhou Medical University, No. 28 Guiyi Street, Yunyan District, Guiyang, 550000 Guizhou China; 2 Institute of Pediatric Research, Children’s Hospital of Soochow University, No.92 Zhongnan Street, SIP, Suzhou, 215003 China; 3 Department of Hematology, Children’s Hospital of Soochow University, No.92 Zhongnan Street, SIP, Suzhou, 215003 Jiangsu China; 4 Department of Traditional Chinese Medicine, Children’s Hospital of Soochow University, Suzhou, 215003 China; 5 Children’s Hospital of Soochow University, Suzhou, 215003 China; 6 Department of Pediatrics, Suzhou Hospital of Anhui Medical University, Suzhou, 234000 China; 7 Department of Pediatrics, The First Affiliated Hospital of Bengbu Medical University, Bengbu, 233004 China; 8 Department of Pediatrics, The Affiliated Hospital of Xuzhou Medical University, Xuzhou, 221000 China; 9 Pediatric Hematology & Oncology Key Laboratory of Higher Education Institutions in Jiangsu Province, Suzhou, 215003 China

**Keywords:** IRF1, AML, Core transcriptional regulatory circuit, Super-enhancer, Lipid metabolism

## Abstract

**Background:**

Acute myeloid leukemia (AML) is a prevalent malignancy of the hematologic system. Despite advancements in therapeutic approaches, significant heterogeneity and therapeutic resistance pose substantial challenges to treatment. Tumors driven by core transcription factors through super-enhancers can establish core transcriptional regulatory circuits (CRCs) that modulate oncogene expression programs. Identifying CRC is crucial for understanding disease-related transcriptional regulation. This study sought to predict and establish a CRC model for AML, identify genes critical for AML survival and explore their regulatory mechanisms in AML progression.

**Methods:**

The dbCoRC tool was used for predictive analysis of H3K27ac ChIP-seq data from 11 AML samples to construct and validate the CRC model in AML patients. To elucidate the functional role of the CRC member IRF1, we utilized short hairpin RNA (shRNA) to knock down IRF1 in AML cells. RNA-seq, CUT&Tag and lipidomics technologies were subsequently used to investigate the regulatory roles and downstream mechanisms of IRF1 in AML.

**Results:**

This study established a core transcriptional regulatory circuit consisting of IRF1, ELF1, ETV6, RUNX2, and MEF2D, which formed an interconnected autoregulatory loop. Further investigations revealed up-regulated expression of IRF1 in AML patients, which was associated with poor prognosis. Inhibition of IRF1 expression resulted in decreased AML cell proliferation and induced apoptosis, indicating its essential role in the survival of AML cells. Additionally, this study revealed that IRF1 directly regulates the transcription of key genes such as FASN, SCD, and SREBF1 for lipid synthesis, thereby affecting lipid metabolism in AML cells.

**Conclusion:**

In summary, this study identified IRF1 as a novel core transcription factor involved in AML pathogenesis. IRF1 collaborates with ELF1, ETV6, RUNX2, and MEF2D to form a core transcriptional regulatory circuit that promotes AML progression. Furthermore, we demonstrated that IRF1 directly regulates the expression of key genes involved in lipid metabolism, influencing the synthesis of diverse lipid molecules crucial for AML survival.

**Supplementary Information:**

The online version contains supplementary material available at 10.1186/s40164-025-00612-z.

## Introduction

Acute myeloid leukemia (AML) is a malignant hematologic disorder characterized by the abnormal proliferation of blast cells in the bone marrow [1]. This condition results in a differentiation defect of the blasts, leading to bone marrow hematopoietic dysfunction and clinical symptoms such as anemia, bleeding tendency, and susceptibility to infection [2]. In recent years, advancements in molecular biology and genomics have led to improved prognoses for AML patients. The 5-year survival rate of AML patients in the United States has increased from 18% in 2000 to 30.5% in 2022 [3]. Despite progress in therapeutic approaches, high heterogeneity and treatment resistance remain major challenges for AML treatment. Therefore, gaining a deeper understanding of the molecular mechanisms and exploring new therapeutic targets are crucial for improving treatment outcomes and increasing survival rates among AML patients.

Super enhancers (SEs) are a distinct class of enhancers that are essential in defining the identity of cells. Compared with typical enhancers (TEs), SEs are enriched with transcription factors (TFs) binding sites, which can attract more TFs and cofactors, leading to the high expression of key cell identity genes. The identification of SEs commonly relies on the presence of H3k27ac and H3K4me1 [4, 5]. SEs play a crucial role in determining the fate decisions of normal cells by regulating the expression of cell type-specific genes [6]. Furthermore, SEs are often aberrantly active in cancer, resulting in the overexpression of oncogenes that promote tumor development [7]. Overall, SEs regulate a wide range of biological processes by modulating the expression levels of many genes and serve as indispensable regulatory elements in cell function and tissue development.

Recent studies have revealed that a cluster of transcription factors, known as core transcription factors, are present in specific cells or tissues. These TFs, regulated by SEs, form a cross-regulatory core transcriptional regulatory circuit (CRC). These CRC genes not only control their own target genes but also regulate each other [8]. In hematological disorders, several CRCs have been identified. For example, Sanda et al. discovered an interconnected positive feedback autoregulatory loop involving TAL1, GATA3, and RUNX1 that promotes the survival of acute T-lymphoblastic leukemia cells [9], while Ott et al. identified a PAX5-dominated regulatory circuit in chronic lymphocytic leukemia [10]. Targeting TFs remains challenging, however, SEs offer opportunities for targeted therapy in cancer cells. Research on BET inhibitors has shown their ability to selectively disrupt the interaction between BET proteins and SEs, thereby inhibiting the oncogenic pathways driven by these SEs [11]. This targeted approach underscores the promising role of BET inhibitors in the development of novel cancer treatments that exploit SE-mediated gene regulation. Our previous study identified 200 SE-regulated genes in 11 AML samples; some of these genes, such as LYL1 and ANP32B, have been shown to be significant in the development of AML [4, 12, 13]. Herein, we aimed to further explore the interaction of SE-regulated TFs and CRCs in AML pathogenesis.

Interferon regulatory factor 1 (IRF1) is a transcription factor that belongs to the interferon regulatory factor family [14]. Initially, identified for its role in inflammation, IRF1 has been implicated in promoting tumor growth and progression. In hepatocellular carcinoma and colon cancer, IRF1 has been shown to suppress the T-cell immune response and facilitate tumor immune escape by directly binding to the promoter of the programmed death ligand 1 (PD-L1) gene, thereby enhancing its transcription [15, 16]. Additionally, IRF1 has been found to decrease the expression of the CXCL9 chemokine gene in breast cancer, leading to inhibition of the antitumor immune response [17]. Notably, high levels of IRF1 expression in glioblastoma are associated with tumor progression and poor prognosis [18]. Despite these findings, the potential role of IRF1 in AML remains unknown. This study identified IRF1 as a novel member of the core regulatory circuitry of AML, where it forms a CRC with other AML core TFs to regulate AML progression.

## Methods

### Cell culture

The human AML cell lines MOLM16, Kasumi-1, HL60, NB4, MV4-11, U937, MOLM13, THP1, HEL, CMK, M07E, Meg01, and K562 were purchased from the National Collection of Authenticated Cell Cultures in Shanghai, China within 5 years. They were all grown in RPMI 1640 medium (VivaCell, China) supplemented with 10% fetal bovine serum (Biochannel, China) and 1% penicillin–streptomycin (Beyotime, China). HEK293FT cells were obtained from the cell bank of the Chinese Academy of Science and cultured in DMEM medium (VivaCell, China) supplemented with 10% fetal bovine serum (Sigma-Aldrich, Germany) and 1% penicillin–streptomycin (Beyotime, China). All the cell lines were verified via short tandem repeats (STR) analysis.

### ChIP-Seq data collection, analysis, and PCA

We conducted a comprehensive analysis of ChIP-Seq datasets derived from 11 AML patient samples, 3 AML cell lines, and 7T-ALL cell lines. The raw data for these datasets, sourced from both our previous work and public databases (with the corresponding GSE numbers detailed in Supplementary Table 1), were aligned to the UCSC hg38 reference genome using Bowtie2 (version 2.3.5) with specific parameters (-p 4 for parallel processing, -q for quiet mode, and -x for indexing the reference genome). Peaks were identified within the aligned data using MACS2 (version 2.0.9) with parameters (-g hs for genome size, -n test for output file naming, -B for bedGraph format output, and -q 0.01 for q-value threshold for peak calling). The resulting bigwig files were visualized using the Integrative Genomics Viewer (IGV) and the Washington University Epigenome Browser. Additionally, bam and narrowPeak files of each H3K27ac ChIP-seq sample were utilized for principal component analysis (PCA) using the R package DiffBind.

### CRC modeling process

The dbCoRC tool [19] was used for predictive analysis of H3K27ac ChIP-seq data from 11 AML samples to construct and validate the CRC model in AML patients. DNA binding motifs for TFs were obtained using the TRANSFAC database and MEME suite. ROSE-identified SE regions were extended 500 bp on both sides, and then motif scanning was performed using FIMO. Self-regulated TF was defined, if a SE-associated TF had more than two binding motifs in its extended SE region. All possible CRCs were then constructed and scored in a given sample. For each candidate CRC, its score was calculated by dividing the total occurrence times of core TFs in all possible circuitries by the number of core TFs in that circuitry.

### Lentiviral preparation and shRNA-mediated knockdown of IRF1, ELF1, ETV6, RUNX2 and MEF2D

The shRNA was synthesized and constructed into the pLKO.1 vector by IGE Biotechnology Ltd, China. HEK293FT cells seeded in 10 cm dishes were transfected with 7.5 μg of purified plasmids together with packaging plasmids, 5.625 μg of psPAX2 and1.875 μg of pMD2.G (Cambridge Biologics, USA), using PEI (Sigma-Aldrich, USA) reagent according to the manufacturer’s protocol. After cultivating for 48 h, the viral supernatant was collected and used to infect cells. Infected cells were incubated with lentivirus for 24 h. Subsequently, the culture medium was exchanged with fresh complete 1640 medium supplemented with 1 ug/ml puromycin (Beyotime, China) to select out the infected cells. IRF1, ELF1, ETV6, RUNX2, and MEF2D knockdown cells were collected after three days of selection and used for subsequent experiments. Detailed shRNA sequence information is listed in Supplementary Table 2.

### RNA extraction and quantitative real-time PCR (qRT-PCR)

Total RNA from the cellular samples was extracted using the Fast Pure Cell/Tissue Total RNA Isolation Kit V2 (Vazyme, China) according to the manufacturer’s instructions. Next, 2000 ng of total RNA was reverse transcribed to cDNA using Prime Script RT Master Mix (Takara, Japan). Then, qRT-PCR analyses were conducted using a LightCycler 480 Real-Time System (Roche, Switzerland). The relative expression levels of each gene were determined on the basis of the average of three replicates. The primer sequences are listed in Supplementary Table 3.

### Western blot analysis

Collected cells were washed with precooled PBS and then lysed with RIPA lysis buffer (Beyotime, China). Western blot was performed using 20 μg of protein separated by SDS-PAGE f and subsequently transferred to a polyvinylidene fluoride membrane (Millipore Sigma, USA). The membranes were incubated with primary antibodies overnight at 4℃. The secondary antibodies were incubated for 1 h at room temperature. Finally, the enhanced chemiluminescence (ECL) solution (Millipore Sigma, USA) was used for visualization using an AI600 image gel imaging analyzer (GE Healthcare Life Sciences, USA). The antibody information used in this study is listed in Supplementary Table 4.

### Co-immunoprecipitation

Co-immunoprecipitation isolated Protein complexes using MAg25K/Protein A/G magnetic beads (Enriching, China). Briefly, MV4-11 cell lysis products were first prepared while MAg25K/Protein A/G magnetic beads were activated. The preactivated beads were subsequently added to the cell lysates and incubated overnight at 4 ℃ following the addition of the primary antibody. The next day, after extensive washing to remove non-specific binding proteins, the immune complexes were eluted from the beads and used for Western blot analysis.

### Cell proliferation and colony formation assays

Cells were selected with puromycin and seeded in 96-well plates at a density of 1 × 10^3^ cells/well, and 100 μl of 1640 medium was added to each well. Cell proliferation was measured on the 1st, 2nd, 3rd, 4th, 5th, and 6th days using a cell counting kit (APExBIO, USA). Knockdown or control cells were seeded in a soft AGAR medium and grown at 37 °C in a humidified environment with 5% CO_2_. After 2 weeks, the cells were fixed with 4% paraformaldehyde, and then stained with Giemsa (Beyotime, China), and the number of colonies was counted.

### Cell cycle analysis

Cells were washed with precooled 1 × PBS and then fixed overnight in 70% ethanol. The next day, the cell cycle distribution was assessed using a Cell Cycle and Apoptosis Analysis Kit (Beyotime, China) following the manufacturer’s instructions. Flow cytometry was used to determine the cell cycle distribution using a Gallios™ flow cytometer (Beckman Coulter, USA). Finally, FlowJo was used to analyze the results.

### Cell apoptosis assay

Cells were first washed with precooled 1 × PBS. The samples were subsequently suspended in Annexin V binding buffer and labeled with FITC-Annexin V antibody and PI solution following the FITC-Annexin V apoptosis kit manual (BD Biosciences, USA). Cell apoptosis was detected by Gallios™ flow cytometer (Beckman Coulter, USA), and the results were analyzed by FlowJo.

### Dual-luciferase reporter assay

The IRF1 SEs were cloned into the pGL3 plasmid. 1 ug plasmid containing the SE sequence or the control plasmid was transfected into MV4-11 cells, along with 50 ng Renilla luciferase plasmid as a normalization control. This transfection was carried out using Lipofectamine LTX reagent obtained from Thermo Fisher, USA. At 48 h post-transfection, luciferase activity was evaluated using the Dual-Luciferase Reporter Assay System (Promega, USA). To account for variations in the enhancer segments present in the transfected cells, the calculated ratio of firefly to Renilla luciferase activity was normalized. The sequence information for these enhancers is shown in Supplementary Table 5.

### CRISPR-Cas9

To generate cell lines with stable gene knockout using the CRISPR/Cas9 system, lentiviral vectors carrying the Cas9 gene were initially transduced into MV4-11 cells. Stable transductants were then selected using geneticin. The plasmid Lenticrispr-copGFP, carrying sgRNA targeting the enhancer regions, was designed and synthesized by GeneChem, China. Subsequently, the Cas9-expressing MV4-11 cells were transfected with either E1-sgRNA or non-targeting control sgRNA (Ctrl-sgRNA) lentiviral particles. The transfection efficiency was assessed by measuring the fluorescence intensity. The sequence information for the sgRNAs targeting these enhancer regions is provided in Supplementary Table 6.

### In vivo experiment

To generate the luciferase-expressing MV4-11 and Kasumi-1 cells, we first constructed a plasmid overexpressing luciferase. This plasmid was then used to package viruses in 293FT tool cells, and subsequently used to transfect MV4-11 and Kasumi-1 cells. After selection with geneticin, stable luciferase-expressing MV4-11 and Kasumi-1 cells were obtained. Five to six-week-old NSG mice were randomly divided into two groups. These luciferase cells were transfected with sh-NC or sh-IRF1 and then injected into the tail vein with 1 × 10^6^ cell suspensions in 200 μl of PBS for each mouse. After several days, intraperitoneal injections of D-luciferin sodium salt (GoldBio, USA) were administered to the mice. Subsequently, under anesthesia, the leukemia burden was visualized using a Berthold imaging system (Berthold Technologies, Germany). After euthanasia by CO2 inhalation, bone, liver, and spleen were collected for further experiments, including the detection of CD45-positive cells, hematoxylin–eosin (HE) staining, and immunohistochemical (IHC) analysis.

### RNA‑seq analysis and data processing

RNA-Seq analysis was detected by Novogene Bioinformatics Technology Co., Ltd. (Beijing, China). The quantification of gene expression was performed using the fragments per million mapped reads per kilobase of exon model (FPKM) metric. Differentially expressed genes (DEGs) were identified using DESeq2 analysis (|log2FoldChange|> 0.5 and p < 0.05). Enrichment analysis was conducted using GSEA software, which was jointly developed by UC San Diego and the Broad Institute, to obtain a deeper understanding of the biological processes linked to the DEGs.

### Immune infiltration analysis

We performed immune infiltration analysis utilizing the Sangerbox online tool [20]. A uniformly normalized pan-cancer dataset, encompassing TCGA TARGET GTEx (PANCAN, N = 19,131, G = 60,499), was downloaded from the UCSC database (https://xenabrowser.net/). Expression data for the IRF1 gene (ENSG00000125347) were extracted. Expression values were log2-transformed [log2(x + 0.001)]. Gene expression profiles were mapped to GeneSymbols, and stromal, immune, and ESTIMATE scores were calculated using the ESTIMATE R package (v 1.0.13) [21]. Pearson correlation coefficients were computed to assess the relationship between IRF1 expression and immune infiltration across tumor types using the corr.test function from the psych R package (v 2.1.6).

### CUT&Tag

Human MV4-11 and Kasumi-1 cells were used for CUT&Tag detection using the Illumina Hyperactive Universal CUT&Tag assay Kit (Vazyme, China) according to the kit instructions. All of the CUT&Tag libraries were sequenced in PE150 mode (SHBIO, China) using an Illumina NovaSeq 6000 platform. The raw data from the CUT&Tag experiments were aligned to the UCSC hg38 reference genome using Bowtie2 (v 2.4.1). Peaks representing regions of interest were identified using MACS2 (v2.0.10). The peaks identified by CUT&Tag analysis are shown in Supplementary Table 7–16.

### Public Hi-C data collection and analysis

Public HiC dataset of MV4-11 cell line (GSE147123) [22] was visualized using the WashU Epigenome Browser (http://epigenomegateway.wustl.edu/browser/).

### HCS LipidTOX™ staining

AML cells were treated with 4% paraformaldehyde for 15 min to fix them. Following washing with precooled PBS, the cells were then incubated with HCS LipidTOX™ green neutral lipid stain (Thermo Fisher, USA) for 30 min at room temperature. DAPI (Invitrogen, USA) was used to counterstain the cell nuclei. Imaging was performed using a fluorescence microscope (Olympus, USA), and the lipid droplet content in the cells was detected via a Gallios™ flow cytometer (Beckman Coulter, USA).

### Lipidomics and data analysis based on LC–ESI–MS/MS system

The MV4-11 cells transfected with sh-NC and sh-IRF1 were collected, rapidly frozen in liquid nitrogen, and then dispatched to Wuhan Mavie Metabolism Biotechnology Co., Ltd., where they underwent comprehensive targeted lipidomics sequencing. The lipidomics data were acquired using a high-resolution LC–ESI–MS/MS system, consisting of an Ultra Performance Liquid Chromatography (UPLC) instrument (ExionLC AD, SCIEX) interfaced with a Tandem Mass Spectrometry (MS/MS) detector (QTRAP® 6500 + , SCIEX). Qualitative analysis was performed based on retention times (RT) and precursor/product ion pairs using a self-built database (MWDB, metware database). Lipid quantification was achieved through Multiple Reaction Monitoring (MRM) mode in the triple quadrupole mass spectrometer. In MRM, the precursor ions (parent ions) of target lipids were first selected, and subsequent fragment ions were filtered to identify specific fragment ions, ensuring precise and reproducible quantification.

After acquiring the lipid mass spectrometry data from different samples, peak areas were integrated for all detected lipids. Integral corrections were applied to normalize the peak intensities of the same lipid across different samples [23]. Initially, missing values in the raw data (ALL_sample_data_raw.xlsx) were filled with 1/5 of the minimum value for each lipid. Subsequently, the coefficient of variation (CV) for quality control (QC) samples was calculated, and only lipids with a CV less than 0.3 were retained in the final data file (ALL_sample_data.xlsx). For detailed lipidomic results, please refer to Supplementary Table 17–19.

### Statistical analysis

All of the experiments were independently conducted at least three times. Statistical analyses were performed using GraphPad Prism 9.0 (GraphPad Software Inc., USA). Student's t-test or the Mann–Whitney U test was used to compare data between two groups. Survival analysis was performed via the Kaplan‒Meier method, and the results were compared via the log-rank test. P values less than 0.05 were regarded as statistically significant (*p < 0.05, **p < 0.01, ***p < 0.001, ****p < 0.0001).

## Results

### Identification of core transcriptional regulatory circuits in AML

**Fig. 1:**
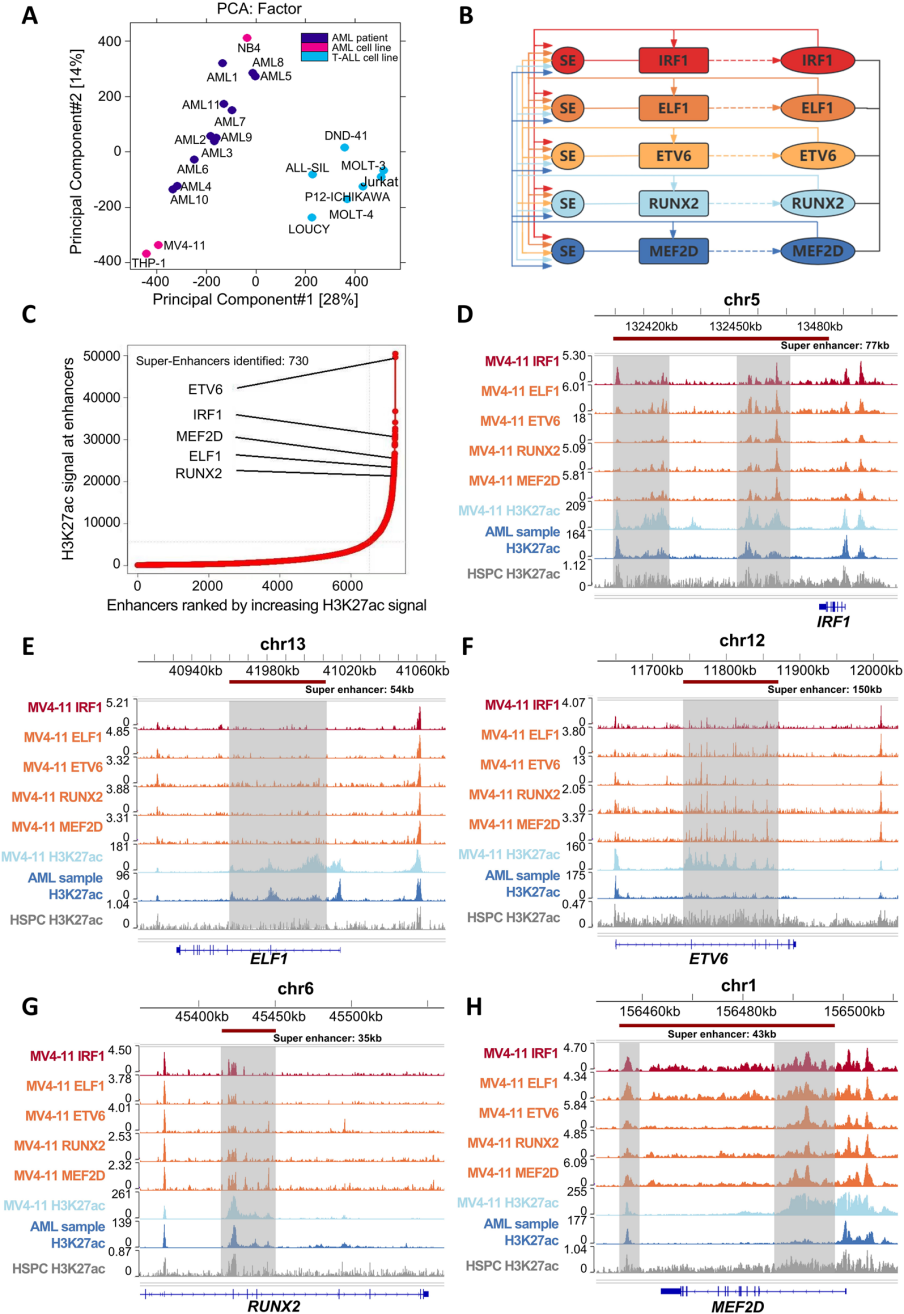
Identification of CRC in AML. **A** PCA of 11 AML samples, 3 AML cell lines, and 7T-ALL cell lines based on H3K27ac identified in each sample. Each circle represents a sample and each color represents the type of sample. **B** Schematic representation of the regulatory relationships of IRF1, ELF1, ETV6, RUNX2 and MEF2D. Rectangles and ovals represent enhancer elements and proteins. **C** Inflection plot showing SE intensity, the SE of AML2 is shown here as an example. **D**–**H** The IGV plots of CUT&Tag and ChIP-Seq data show SE peaks (shaded regions) for ELF1, ETV6, RUNX2, and MEF2D near their respective genes

We conducted an analysis of H3K27ac ChIP-seq data obtained from 11 patients diagnosed with AML (clinical information for these patients is provided in Supplementary Table 20), as well as data from 3 AML cell lines and 7T-ALL cell lines (Supplementary Table 1). The results of PCA and clustering using peak signals effectively differentiated between the AML and T-ALL cell lines (Fig. 1A and Supplementary Fig. 1A). We subsequently used the dbCoRC tool to developed a CRC model for AML patients, incorporating several classic transcription factors in AML such as ETV6, RUNX2, MEF2D, IRF1, and ELF1 [19, 24, 25]. (Fig. 1B, Supplementary Fig. 1B and Supplementary Table 21). Figure 1C and Supplementary Fig. 2 demonstrate that these five TFs were regulated by SEs in samples from AML patients. To validate the interactions among these five TFs, we performed CUT&Tag in MV4-11 cells to map the genome-wide binding signals of the aforementioned five TFs. As anticipated, the peaks of SEs are located near genome region of the transcription factors IRF1, ELF1, ETV6, RUNX2, and MEF2D (Fig. 1D–H and Supplementary Fig. 1C-G). Furthermore, we integrated H3K27Ac ChIP-Seq data from MV4-11 cells and AML patients with normal hematopoietic stem and progenitor cells (HSPCs) serving as negative controls. ChIP-Seq analysis revealed that these five TFs are regulated by SEs in both AML patients and AML cell lines, indicating that their expression is elevated specifically in AML. Conversely, in normal HSPCs, the H3K27ac modification associated with these TFs was notably weak, suggesting their specific activation in AML (Fig. 1D–H and Supplementary Fig. 1C-G). Collectively, these data indicated that we identified an AML-specific CRC in AML consisting of IRF1, ELF1, ETV6, RUNX2, and MEF2D.

### The five TFs in the CRC regulate and influence each other's transcription

**Fig. 2:**
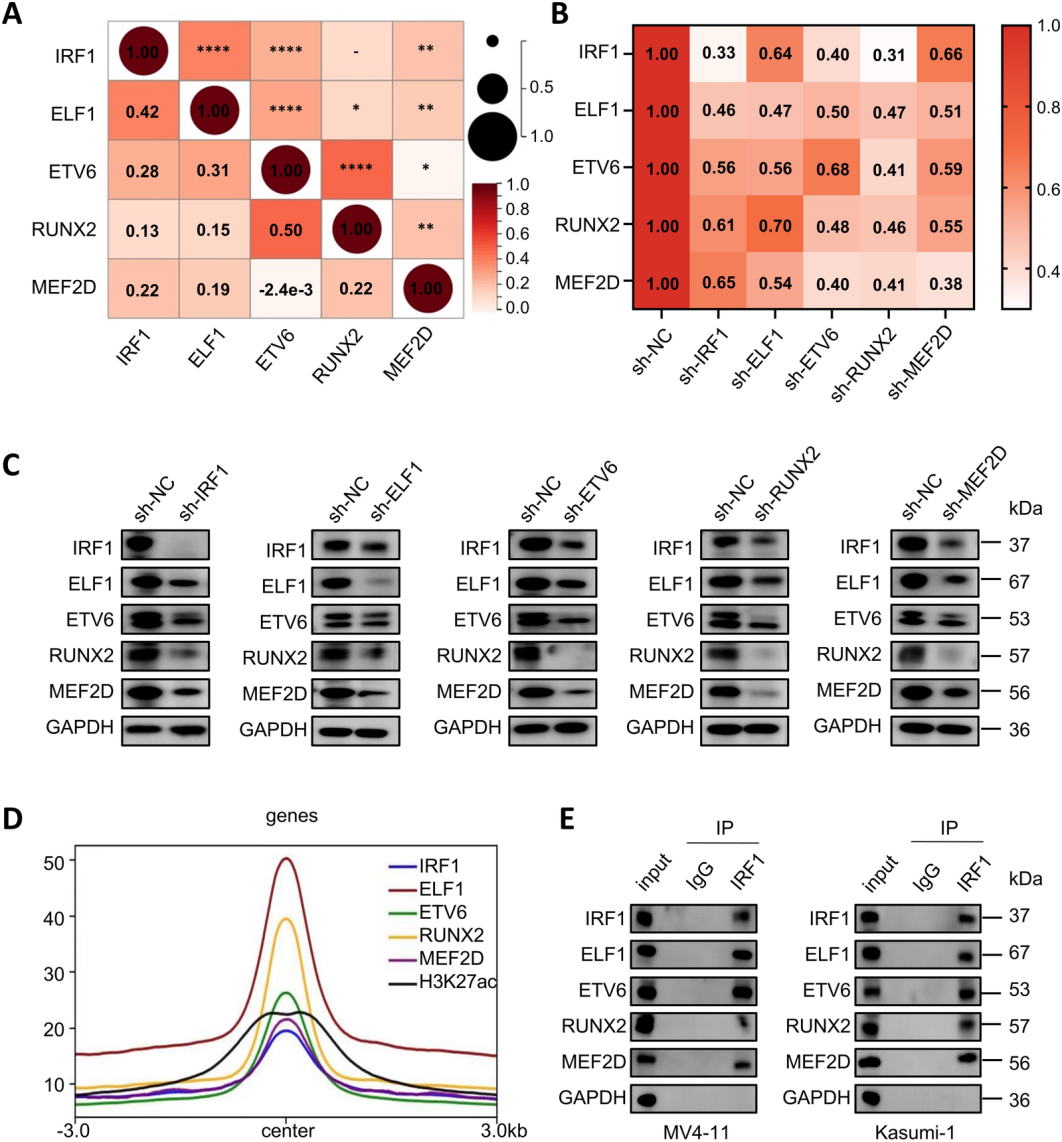
IRF1, ELF1, ETV6, RUNX2, and MEF2D are mutually regulated and affect each other's transcription. **A** Heatmaps were generated by the SangerBox tool to depict the Pearson correlation coefficients between the five master TFs in the TCGA database of AML patients (n = 187). **B** Heatmap of each master TF detecting changes in mRNA levels of the remaining transcription factors after a knockdown by shRNA. **C** Western blot of each TF after knockdown by shRNA to detect changes in protein levels of the remaining transcription factors. **D** Line graph showing the distribution of CUT&Tag signals in the MEF2D peak region for the main 5 TFs. **E** IRF1 immunoprecipitation successfully captured ELF1, ETV6, RUNX2 and MEF2D

The expression of TFs in the CRC is typically positively correlated [26]. To investigate this, we analyzed the TCGA dataset and generated a correlation heatmap using the Sanger box [20], which revealed significant positive correlations in the expression of these five genes in AML patients (Fig. 2A). Subsequent knockdown experiments in MV4-11 cells demonstrated that down-regulation of any of the TFs led to decreased expression of the other four members at both the mRNA and protein levels, confirming their inter-regulation (Fig. 2B, C). Furthermore, metagene analyses [27] revealed a high degree of overlap in the distribution of binding peaks for the five TFs (Fig. 2D and Supplementary Fig. 3A-D). Additionally, immunoprecipitation with an IRF1 antibody revealed that IRF1 could bind to ELF1, ETV6, RUNX2 and MEF2D in MV4-11 and Kasumi-1 cells (Fig. 2E). In summary, these results validated the interaction among the five TFs involved in AML-CRC.

### Downregulation of IRF1 inhibits AML cell proliferation and promotes apoptosis in vitro

Analysis using the GEPIA database [28] and TCGA dataset revealed that these five genes were significantly overexpressed in AML samples compared with normal samples, and their expression levels were correlated with poor prognosis of AML patients (Supplementary Fig. 4). ETV6, RUNX2, and MEF2D are established oncogenes for AML development [29–32], while the roles of IRF1 and ELF1 in AML are still unclear. Therefore, we sought to investigate the functions of IRF1 and ELF1 in AML cells. Compared with the downregulation of ELF1 (Supplementary Fig. 5), we observed that the downregulation of IRF1 significantly inhibited the survival of MV4-11 and Kasumi-1 cells. Consequently, our focus shifted toward further study of IRF1.

**Fig. 3:**
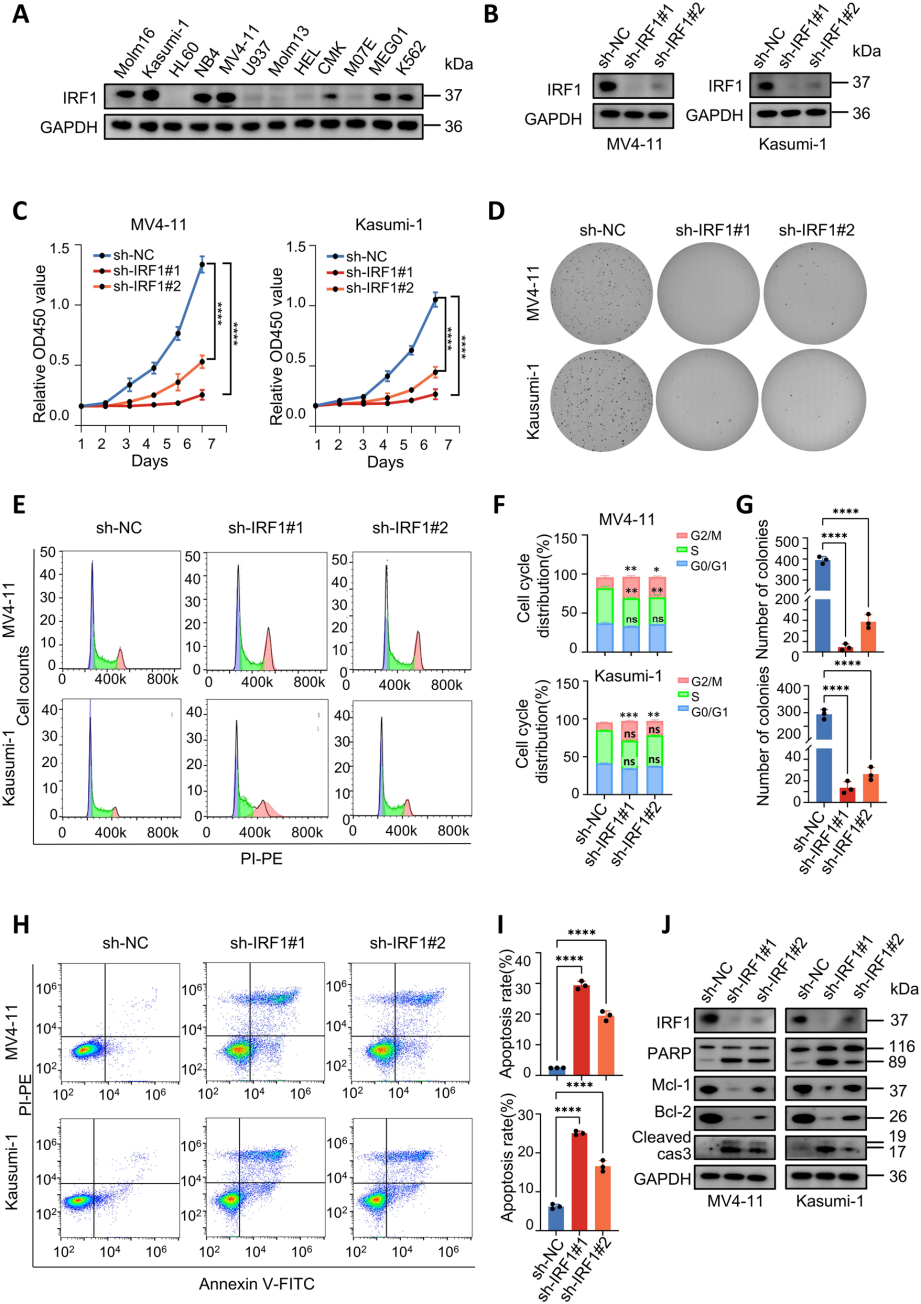
IRF1 is a gene important for AML cell survival. **A** Protein expression levels of IRF1 in AML cell lines. **B** Western blot was used to verify the knockdown efficiency of IRF1 in AML cells. **C** CCK8 detection of AML cell proliferation after infection with sh-NC or sh-IRF1. **D** Colony formation assay of sh-NC or sh-IRF1 infected MV4-11 and Kasumi-1 cells. **E**, **F** Cell cycle detection by flow cytometry after sh-NC or sh-IRF1 infection of MV4-11 and Kasumi-1 cells. **G** The number of colonies in sh-NC, sh-IRF1#1 and sh-IRF1#2 groups. **H**, **I** Knockdown of IRF1 resulted in an increased apoptosis rate in AML cells. **J** Western blot analysis showed that PARP and Cleaved Caspase3 expression was up-regulated and Mcl-1 and Bcl-2 expression was down-regulated in MV4-11 and Kasumi-1 cells after IRF1 knockdown

Given the crucial role of IRF1 in immune defense, we assessed its immune scores in AML and other types of tumors. These findings revealed a positive correlation between IRF1 expression and immune infiltration in various tumors such as breast cancer, renal carcinoma, and cutaneous melanoma. However, this correlation was not observed in AML, indicating a potentially distinct role of IRF1 in AML compared to other tumor types (Supplementary Fig. 6). Analysis of the GEPIA dataset demonstrated that IRF1 exhibited higher median expression levels in AML compared to other tumor types, with elevated expression levels observed in cancer cells compared to normal cells (Supplementary Fig. 7A). To validate the function of IRF1 at the cellular level, we assessed the expression level of IRF1 in AML cell lines. We observed different expression levels of IRF1 across distinct AML cellular lines **(**Fig. 3A and Supplementary Fig. 7B). Consequently, we selected six cell lines with distinct genetic backgrounds, including MV4-11, Kasumi-1, Molm16, THP1, HEL, and CMK, for subsequent experiments. In comparison with the negative control (sh-NC), shRNA targeting IRF1 effectively reduced the expression of IRF1 in AML cell lines (Fig. 3B, Supplementary Fig. 7C and 8A).

Since IRF1 is a crucial molecule in the virus-activated IFN pathway, we further verified whether the lentiviral transfection process affected the expression of IRF1 by conducting experiments in MV4-11 cells with high IRF1 expression and U937 cells with low expression. The results indicated that lentiviral transfection did not alter the level of IRF1 expression (Supplementary Fig. 7D). Knockdown of IRF1 led to significant cell death (Supplementary Fig. 7E). Furthermore, both the CCK8 cell proliferation assay (Fig. 3C and Supplementary Fig. 8B) and soft agar clone formation assay (Fig. 3D, G and Supplementary Fig. 8C) demonstrated reduced cell proliferation and colony formation capability after IRF1 knockdown. The cell cycle assay results suggested that the cell cycle was predominantly blocked in the G2/M phase (Fig. 3E, F, Supplementary Fig. 8D and 9A), and levels of cell cycle proteins including CDK2, CDK4, and CDK6 were down-regulated in IRF1 knockdown groups (Supplementary Fig. 7F). Additionally, knocking down IRF1 resulted in increased apoptosis in MV4-11 and Kasumi-1 cells, accompanied by upregulation of PARP and cleaved Caspase 3 expression as well as downregulation of Mcl-1 and Bcl-2 expression (Fig. 3H–J and Supplementary Fig. 9B-C). These findings collectively indicate that IRF1 is an essential gene for AML cell survival.

### Downregulation of IRF1 significantly inhibits the progression of AML in vivo

**Fig. 4:**
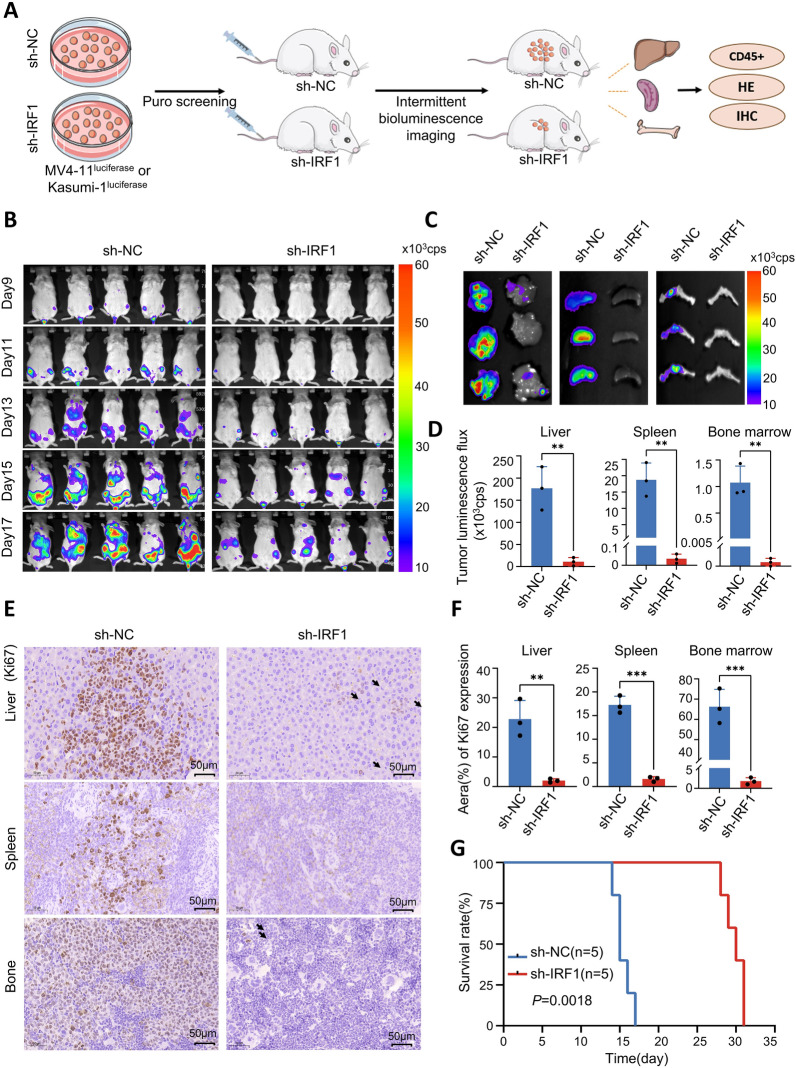
Knockdown of IRF1 inhibits AML proliferation in vivo. **A** Schematic diagram of animal experimental design. **B** Bioluminescence imaging on days 9, 11, 13, 15, and 17 in the IRF1 knockdown group and control group injected MV4-11. **C**, **D** Bioluminescence imaging and statistical analysis of liver, spleen, and bone in IRF1 knockdown group and control group injected MV4-11. **E**, **F** Representative images and statistical analysis of IHC staining (KI67) of liver, spleen, and bone in two groups of mice injected MV4-11. **G** Survival curves of mice in both groups injected MV4-11 (n = 5)

To further explore the impact of IRF1 on AML progression in vivo, we intravenously injected NSG mice with luciferase-labeled MV4-11 or Kasumi-1 cells from sh-IRF1#1 and sh-NC groups (Fig. 4A). As depicted in Fig. 4B and Supplementary Fig. 10A, the bioluminescent signal intensity was markedly reduced in IRF1 knockdown mice compared to the control group. The bioluminescence flux histograms of mice injected with either MV4-11 or Kasumi-1 cells consistently demonstrated that the tumor burden in the sh-IRF1 group was significantly lower compared to that in the sh-NC group (Supplementary Fig. 10D-E). Bioluminescence imaging of mouse liver, spleen, and bone marrow samples revealed substantial decreases in tumor burden following IRF1 downregulation (Fig. 4C, D and Supplementary Fig. 10B-C). However, there were no discernible differences in appearance or weight of the liver and spleen between the two groups (Supplementary Fig. 10F-H). Additionally, survival analysis showed prolonged survival among IRF1 knockdown mice (Fig. 4G and Supplementary Fig. 10I). Furthermore, we assessed the count of human CD45-positive cells in the liver, spleen, and bone marrow of mice from both groups and found that the ratio of human CD45-positive cells was significantly lower in the IRF1 knockdown group compared to the control group (Supplementary Fig. 11A-D). HE staining analysis of mouse liver, spleen, and bone marrow indicated a significant reduction in tumor cells within the IRF1 knockdown group (Supplementary Fig. 11E). IHC staining for Ki67 revealed a notable decrease in Ki67-positive cells within the IRF1 knockdown group (Fig. 4E, F).The aforementioned results confirm that IRF1 knockdown inhibits AML progression in vivo.

### IRF1 is a SE-driven gene in AML, and IRF1-SE plays an important role in IRF1 transcription

**Fig. 5:**
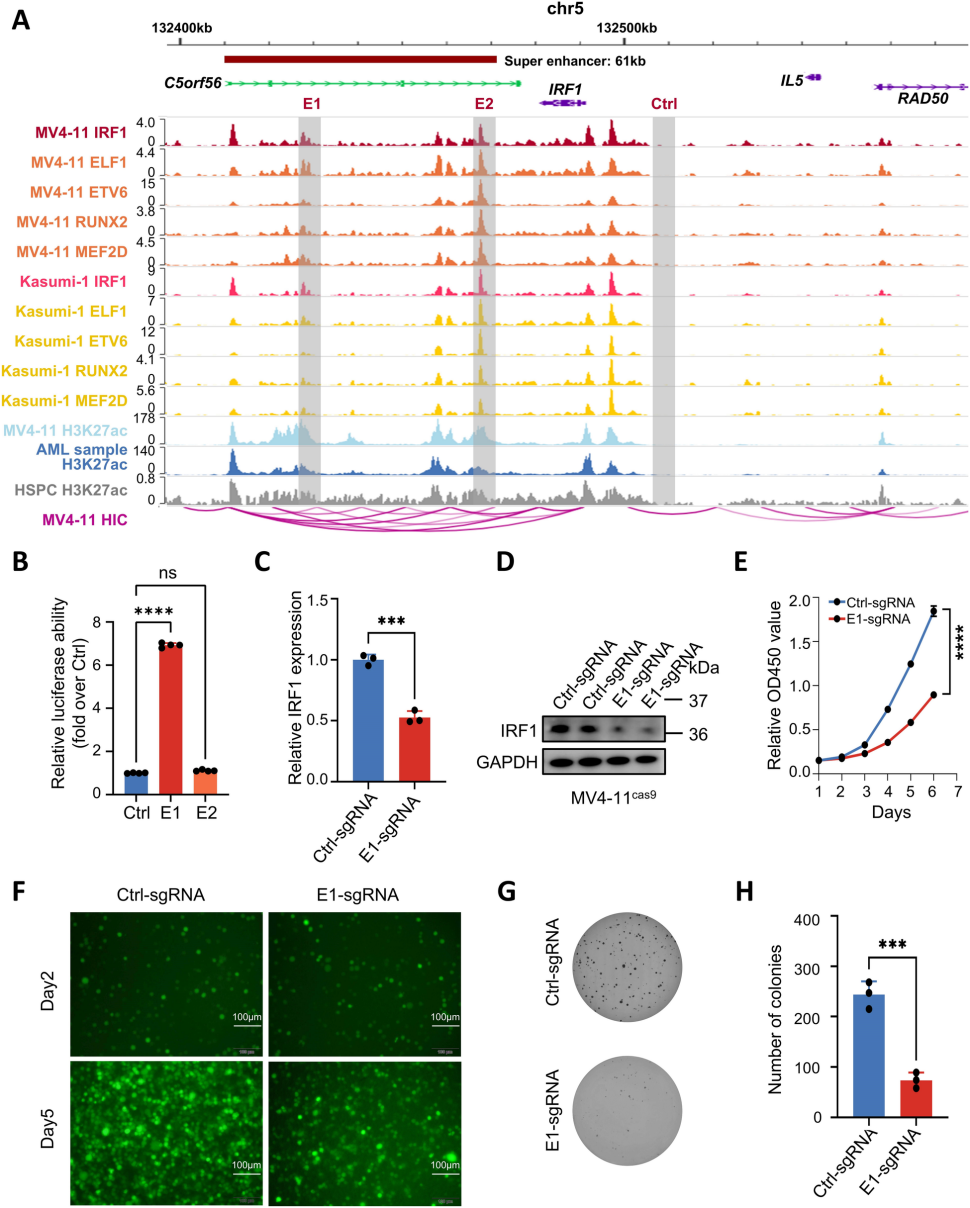
IRF1 is a super-enhancer-driven gene in AML. **A** Interactions between SE and promoter regions using MV4-11 HiC dataset. **B** The 2 enhancer components within IRF1-SE (E1 and E2) as well as the negative control region (Ctrl) were cloned and transferred into luciferase reporter vectors. The activity of these enhancers was assessed by a dual luciferase reporter gene assay performed in MV4-11. **C** Expression of IRF1 at the cellular mRNA level after transfection of MV4-11^cas9^ with Ctrl-sgRNA and E1-sgRNA. **D** Expression of IRF1 at the cellular protein level after transfection of MV4-11^cas9^ by Ctrl-sgRNA and E1-sgRNA. **E** The survival rate of Ctrl-sgRNA and E1-sgRNA after transfection of MV4-11^cas9^ cells was assessed by the CCK8 method to evaluate their cell viability. **F** Fluorescence microscopy showed that knockdown of enhancer sequence E1 significantly reduced the proliferation of MV4-11^cas9^ cells compared with the control. **G**, **H** Specific inhibition of IRF1-E1 cis-regulatory elements on colony formation of MV4-11^cas9^ cells

To further investigate the role of SE in regulating IRF1 expression, we analyzed public HiC dataset of MV4-11 cell line (GSE147123). Our analysis identified two distal enhancers E1 and E2 which potentially interact with the IRF1 promoter (Fig. 5A). Using a luciferase reporter assay, we demonstrated a strong activating effect of E1 on the transcriptional activity of IRF1 (Fig. 5B), leading us to conclude that the E1 element was indeed the main active unit within IRF-SE. To further validate this finding, CRISPR-Cas9 was employed to knock out the E1 region. As depicted in Fig. 5C, D, the sgRNA targeting E1 clearly reduced both the mRNA and protein levels of IRFI. Furthermore, inhibition of IRF1-SE activity attenuated the proliferation and colony formation capabilities of AML cells (Fig. 5E–H). These findings not only underscored the regulatory role of IRF1-SE in controlling IRF1 expression but also highlighted the potential importance of IRF1 in AML cell proliferation.

### Knockdown of IRF1 inhibits AML proliferation via affecting the MYC pathway

**Fig. 6:**
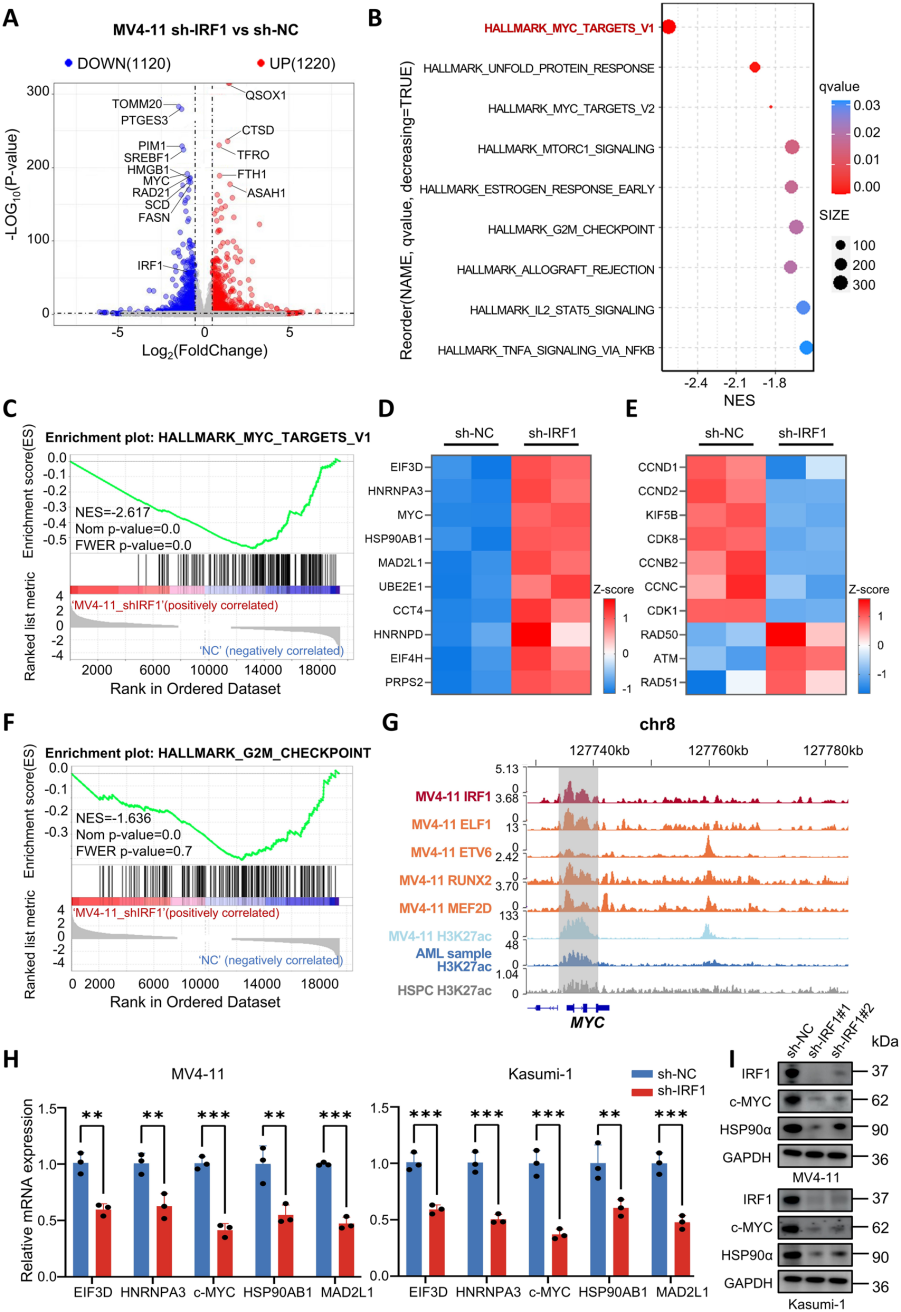
Downregulation of IRF1 expression inhibits the MYC signaling pathway. **A** Volcano plot of differentially expressed genes between IRF1 knockdown and control groups. Red and blue colors indicate up-and down-regulated genes, respectively (|log2FoldChange|> 0.5, p < 0.05). **B** Differential gene KEGG pathway enrichment analysis. **C** GSEA plot showing that IRF1 knockdown-treated MV4-11 cells were enriched in the MYC_TARGETS_V1 pathway. **D** heatmap showing the expression levels of MYC pathway genes in MV4-11 cells after IRF1 knockdown. **E** heatmap showing the expression levels of cell cycle associated genes in MV4-11 cells after IRF1 knockdown. **F**) GSEA plot showing that IRF1 knockdown-treated MV4-11 cells were enriched in the G2M CHECKPOINT pathway. **G** IGV visualization displayed the whole-genome map of the MYC gene. **H** qRT-PCR detection of reduced expression levels of EIF3D, HNRNPA3, c-MYC, HSP90AB1 and MAD2L1 in the MYC pathway after IRF1 knockdown. **I** Western blot detected down-regulation of c-MYC and HSP90α protein expression after IRF1 knockdown

To investigate the potential molecular mechanisms involved in the role of IRF1 in AML, we conducted an RNA sequencing analysis (Supplementary Table 22). The results revealed that 1220 genes were up-regulated and 1120 genes were down-regulated in IRF1 knockdown MV4-11 cells (|log2FoldChange|> 0.5, p < 0.05) (Fig. 6A). GSEA and KEGG analysis indicated that the differentially expressed genes were enriched primarily in the MYC_TARGETS_V1 pathway (Fig. 6B–D, Supplementary Table 23). Furthermore, the G2M_CHECKPOINT pathway was also significantly enriched, which is consistent with the previously observed cell cycle arrest (Fig. 6E, F). IGV visualization demonstrated strong occupancy signals in the promoter region of the MYC gene in IRF1 and other TFs from the AML-CRC (Fig. 6G). Subsequently, qRT-PCR and western blot were performed to validate these findings in MV4-11 and Kasumi-1 cells. As anticipated, the mRNA and protein levels of MYC pathway-associated genes, including c-MYC and HSP90AB1, decreased following IRF1 knockdown (Fig. 6H, I). These observations suggest that the downregulation of IRF1 expression may inhibit AML cell proliferation by affecting the MYC pathway.

### Downregulation of IRF1 affects key genes involved in lipid synthesis and leads to significant inhibition of lipid synthesis in AML cells

**Fig. 7:**
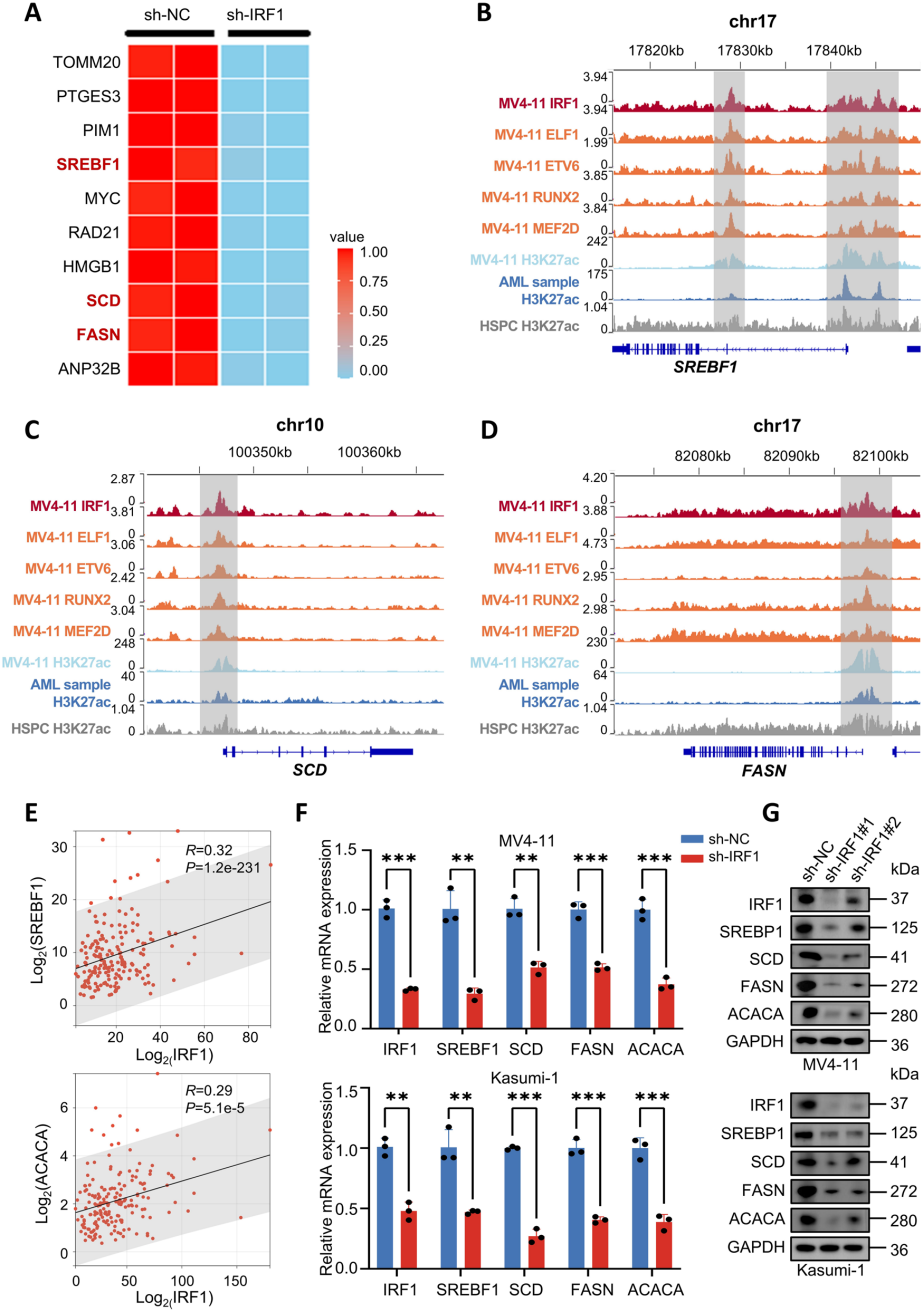
IRF1 downregulation affects the expression of lipid synthesis-related genes. **A** Heatmap showing the top 10 downregulated genes (sorted by p-value) after IRF1 knockdown in MV4-11 cells. **B** IGV visualization of SREBF1 whole-genome map. **C** IGV visualization of SCD whole-genome map. **D** IGV visualization of FASN whole-genome map. **E** Scatter plot showing the correlation between IRF1 and mRNA levels of SREBF1 and ACACA in AML patients in the TCGA database. **F** qRT-PCR is used to detect the reduced expression levels of lipid synthesis-related genes SREBF1, SCD, FASN, and ACACA following the IRF1 knockdown. **G** Western blot detected down-regulation of SREBF1, SCD, FASN, and ACACA protein expression following IRF1 knockdown

Interestingly, three of the top 10 downregulated genes (ranked by p-value) identified via transcriptomic sequencing are crucial for regulating lipid synthesis: SREBF1, SCD, and FASN. These findings suggest that IRF1 may influence AML progression by modulating lipid synthesis (Fig. 7A). IGV visualization showed strong occupancy signals for IRF1 and other AML CRC genes in the promoter regions of SREBF1, SCD, and FASN (Fig. 7B–D), indicating that IRF1 may regulate their transcription by forming CRCs with other TFs. Further analysis of the TCGA dataset revealed a positive correlation between IRF1 and key lipid metabolism genes such as SREBF1 and ACACA (Fig. 7E). The reduced mRNA and protein expression levels of key lipid synthesis genes following IRF1 knockdown were confirmed by the qRT-PCR and western blot, which was consistent with the transcriptomic sequencing data (Fig. 7F, G). These results suggest that IRF1 may affect AML progression by modulating the expression of lipid synthesis-related genes.

Subsequently, we investigated the sensitivity of AML cells to the MYC inhibitor 10058F4 and inhibitors targeting three key lipogenesis-related genes: Orlistat (targeting FASN), Fatostatin (targeting SREBF1), and A939572 (targeting SCD) [33–36]. The results (Supplementary Fig. 12A-C) demonstrate that these inhibitors can effectively reduce the survival rates of AML cells to varying degrees. To explore the potential synergistic effects between MYC and lipid metabolic pathways in AML, we evaluated the combined action of the MYC inhibitor 10058-F4 with three lipid metabolism inhibitors. Drug synergy scores were calculated using response surface modeling and zero interaction potency (ZIP) through the online SynergyFinder software (https://synergyfinder.fimm.fi) [37, 38]. The results indicate that the combination of 10058F4 with Orlistat, Fatostatin, or A939572 exhibits a certain degree of synergism in both cell lines, albeit not to a highly potent extent (Supplementary Fig. 12D).

### The impact of IRF1 on lipid synthesis in AML cells: a mechanistic perspective

**Fig. 8:**
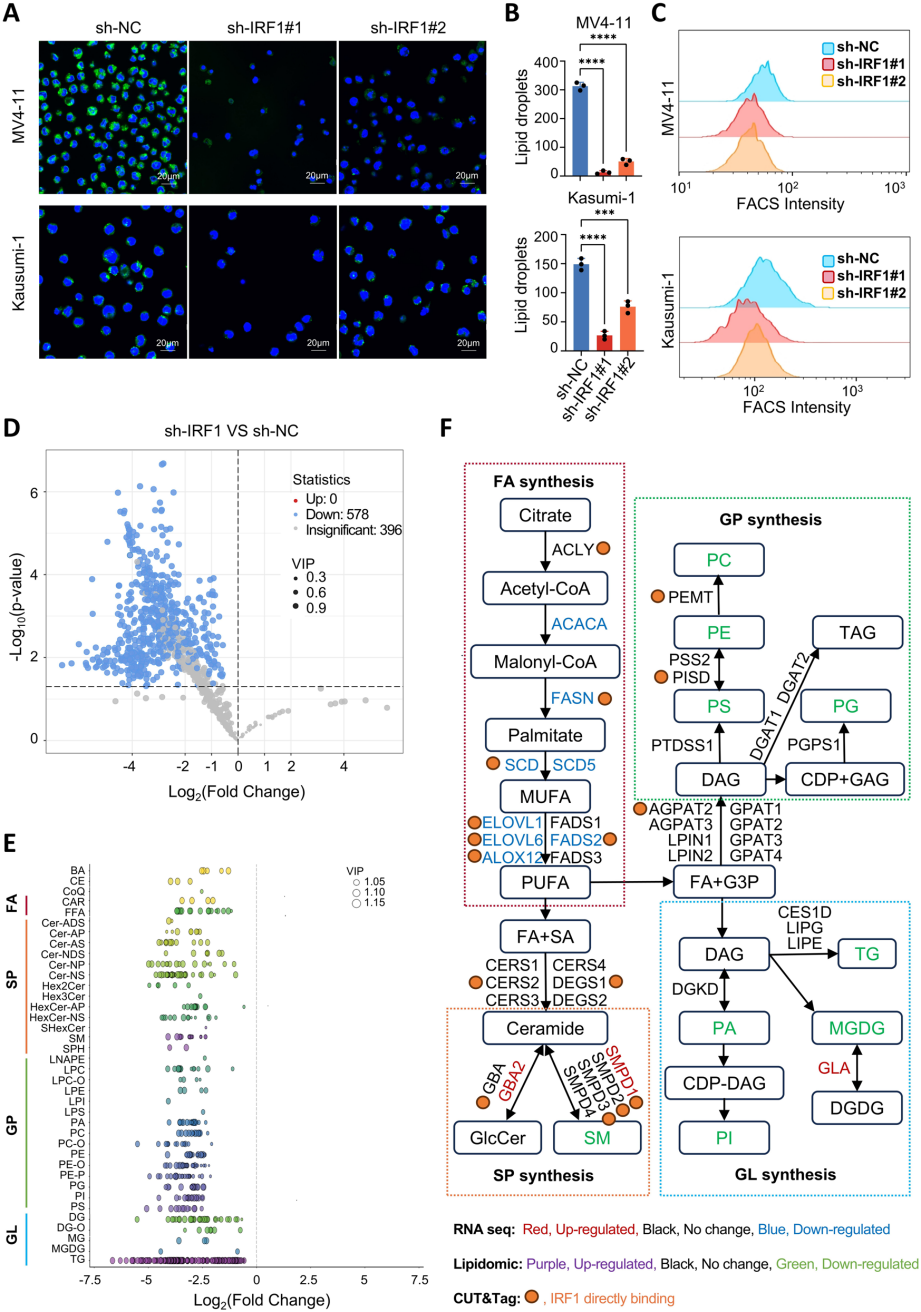
Mechanisms by which IRF1 regulates lipid synthesis in AML cells. **A** Confocal images of lipid droplets in IRF1 knockdown MV4-11 cells. **B** Quantification of lipid droplets in MV4-11 cells following IRF1 knockdown using confocal microscopy. **C** Flow cytometry analysis of lipid droplet content in the two groups. **D** Volcano plots of LC–ESI–MS/MS system-based lipidomics after IRF1 knockdown. Each point represents a lipid. **E** Lipidomic bubble plots showing changes in lipid metabolites in IRF1 knockdown MV4-11 cells. Each point represents a metabolite. **F** Schematic showing IRF1 regulation of lipid synthesis pathways by integrating RNA-seq, CUT&Tag, and lipidomics data

Given the regulatory role of IRF1 in the expression of key lipid synthesis genes, we further investigated its involvement in lipid metabolism in AML. Initially, HCS LipidTOX™ staining was performed on both IRF1 knockdown and control cells, followed by quantification of the lipid droplet content using immunofluorescence and flow cytometry. The results demonstrated a significant reduction in intracellular lipid droplet synthesis following the downregulation of IRF1 (Fig. 8A–C). Subsequently, an extensive targeted lipidomic analysis was carried out to identify changes in lipid types within AML cells after IRF1 knockdown. The findings revealed that the downregulation of IRF1 led to a decrease in 578 different lipid types in AML cells (Fig. 8D and Supplementary Fig. 13).

**Fig. 9:**
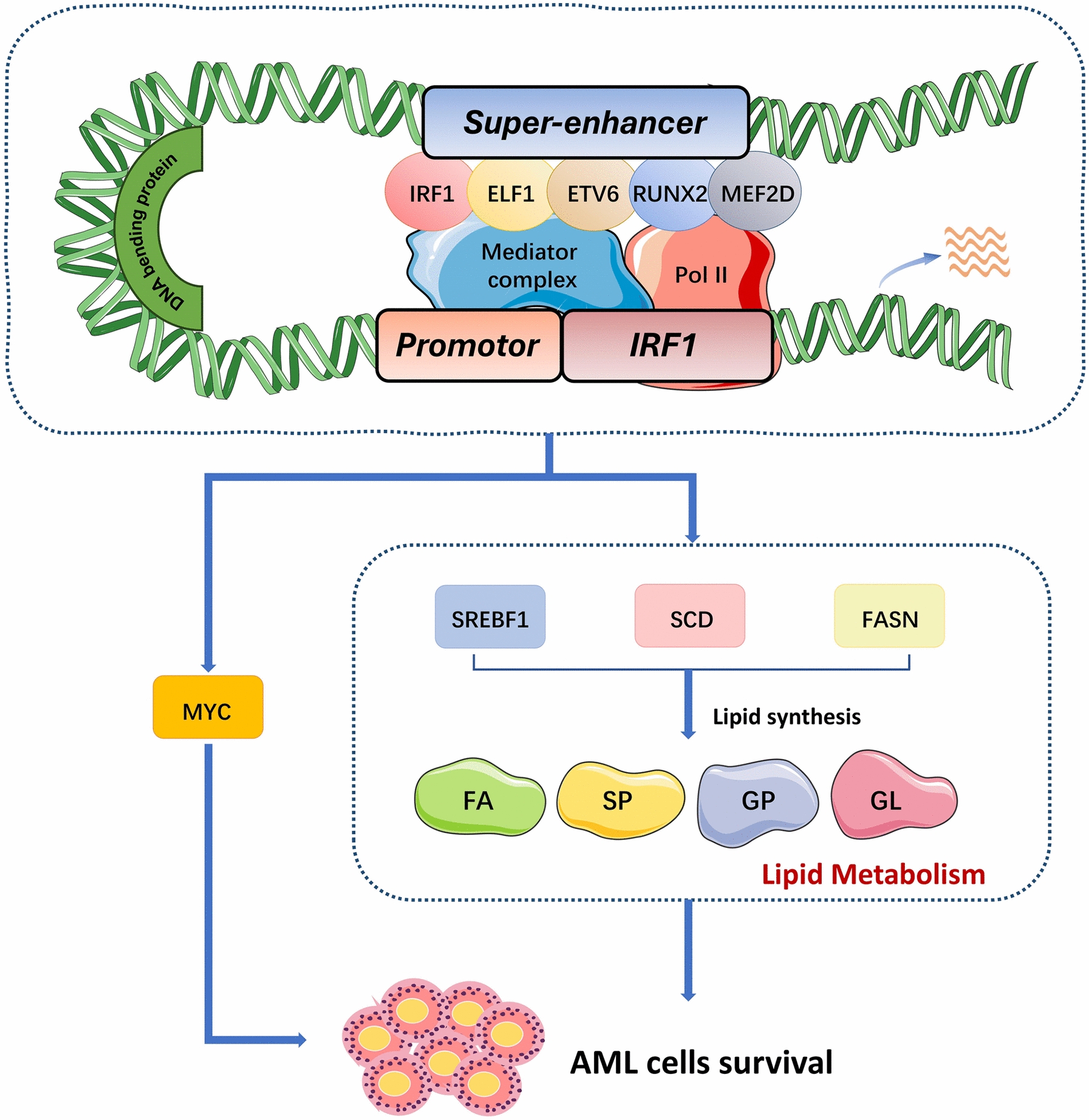
Model depicting the effects of IRF1 in AML cells. IRF1 collaborates with the classical core transcription factors ELF1, ETV6, RUNX2, and MEF2D to regulate AML progression. Mechanistically, IRF1 facilitates the progression of AML by regulating the expression of MYC. Furthermore, IRF1 modulates the expression of key lipogenic genes, including SCD, FASN and SREBF1, thereby promoting lipid synthesis and enhancing AML cell survival

As illustrated in Fig. 8E, the down-regulated lipid molecules were significantly enriched in four types of lipids: (1) fatty acyls (FA), including acylcarnitines (CAR) and free fatty acids (FFA); (2) sphingolipids (SP), including glycosphingolipids (Hex2Cer), sphingomyelin (SM), and sphingomyelin (SPH); (3) glycerophospholipids (GP), such as lysophosphatidylcholine (LPC), lysophosphatidic phosphatidylethanolamine (LPE), and phosphatidic acid (PA); (4) Glycerolipids (GL), such as monoglycerides (MG) and monoglycosyl diglycerides (MGDG). Some of these down-regulated lipids, such as fatty acids, have been identified as critical for tumor cell growth, survival, and drug resistance [39]. Through integrated enrichment analysis of RNA sequencing and lipidomics datasets, we found enrichment of lipid metabolism pathways including sphingolipid metabolism (Supplementary Fig. 14). Thus, IRF1 may regulate AML cell growth and survival by modulating the metabolism of a wide range of lipids. To gain a comprehensive understanding of lipidomics changes after IRF1 downregulation, we integrated the RNA sequencing, CUT&Tag, and lipidomics results (Fig. 8F). In summary, these findings suggest that IRF1 may influence broad lipid biosynthesis in AML cells by directly regulating the expression of key lipid synthesis genes (Fig. 9).

## Discussion

Transcriptional dysregulation plays a direct role in the development and progression of cancer [40]. SEs are key components that help maintain the identity of cancer cells and promote abnormal transcription in these cells [41]. Both normal and malignant cells rely on a cluster of core transcription factors (TFs) to synergistically regulate their transcriptional programs. These TFs directly co-occupy SEs, as well as each other's SEs, forming interconnected core transcriptional regulatory circuits [42]. The aberrant activities of specific TFs and regulatory networks are closely associated with the onset and development of various subtypes of leukemia. Researchers have identified the core regulatory networks for leukemia types such as T-cell acute lymphoblastic leukemia (T-ALL), chronic lymphocytic leukemia (CLL), and mixed-lineage leukemia-rearranged acute myeloid leukemia (MLL-rearranged AML). These findings provide valuable evidence for understanding the pathogenesis of leukemia and identifying potential therapeutic targets [9, 10, 43].

Our study identified super-enhancers in AML patients using H3K27ac and determined a CRC model composed of IRF1, ELF1, ETV6, RUNX2, and MEF2D. These transcription factors promote each other's expression through interactions with their super-enhancers; knocking down any TF disrupts the entire core transcriptional regulatory circuitry program. Among the 5 TFs identified in our AML-CRC model, ETV6, RUNX2, and MEF2D are known as critical genes that promote AML survival. These genes are highly expressed in AML and are correlated with poor prognosis [29–32]. Additionally, we identified 2 genes whose functions have not been clearly reported in AML: IRF1 and ELF1. ELF1 currently plays a regulatory role mainly in autoimmune disorders such as systemic lupus erythematosus, as well as in some cancers [44]. ELF1 can activate MEIS1 transcription to promote glioma progression, and it is also involved in the regulation of the relapsing cell state in melanoma and the phenotypic transition [45, 46]. However, few studies have reported its function in AML. We down-regulated ELF1 expression in AML cells which showed suppressed proliferation and increased apoptosis. GEPIA and TCGA data also demonstrated that ELF1 was highly expressed in AML and correlated with poor prognosis. These results suggest that ELF1 may act as an oncogene to maintain AML progression. The role of ELF1 in AML remains to be further investigated.

IRF1 is the initial member of the interferon regulatory factor family to be identified, and it typically functions as an IFN-stimulated gene (ISG) in IFN-mediated signaling, thereby bolstering the body's innate immune defenses against pathogenic infections [47]. The role of IRF1 in cancer progression is currently a topic of debate, contingent upon the specific type of cancer. While various studies have reported the anti-proliferative role of IRF1, there are also indications that IRF1 contributes to the proliferation and metastasis of tumor cells in cancers such as esophageal cancer and head and neck squamous cell carcinoma [48–50]. Immune infiltration scores suggest a positive correlation between IRF1 and immune infiltration in tumors like breast cancer, renal cancer, and cutaneous melanoma; however, no such correlation exists in AML, indicating that IRF1 may function uniquely in AML compared with other types of tumors. Our data demonstrate that IRF1 acts as an oncogene in AML cells, and that downregulating IRF1 can inhibit AML cell proliferation and induce apoptosis. Research on leukemia has shown that IRF1 is highly expressed in AML stem cells, patient samples, and cell lines, and is correlated with poor prognosis. High expression levels of IRF1 have also been observed in LSCs from drug-resistant patients with CML [51–53]. Taken together, this evidence supports the notion that IRF1 operates as an oncogene specifically within AML.

Our study revealed that the downregulation of IRF1 significantly affected the MYC pathway, leading to a decrease in cell proliferation and cell cycle arrest. The activation of the MYC pathway is known to play a crucial role in promoting proliferative signaling, inhibiting growth inhibition, evading immune responses and cellular metabolic changes, as well as other biological processes in human cancers [54]. Previous research has demonstrated that high expression levels of c-MYC are associated with the progression of AML, and inhibiting c-MYC expression can impede AML progression [55]. Furthermore, our findings indicate that IRF1, ELF1, ETV6, RUNX2, and MEF2D bind to the MYC promoter, suggesting that IRF1 may regulate MYC expression by forming a CRC complex with other transcription factors. Specifically, it has been reported that RUNX2 activates MYC and promotes the development of plasmacytoid dendritic cell neoplasms [56], while further investigation is needed to understand how the other genes regulate MYC.

We also observed significant inhibition of extensive lipid metabolism in AML cells following IRF1 knockdown. Lipid metabolism is a crucial cellular process responsible for converting nutrients into metabolic intermediates used in membrane biosynthesis, energy storage, and the production of signaling molecules [57]. Human tumor cells exhibit active proliferation and significantly enhanced levels of lipid synthesis to meet the demands of enhanced biofilms [58]. Previous reports have indicated that IRF1 is proatherogenic, and its deficiency inhibits lipoprotein uptake, promotes cholesterol efflux, and alters the expression of lipid metabolism-associated genes [59]. However, the regulatory networks of lipid metabolism in AML have not been well-studied. In our study, IRF1 was recognized as a novel regulator of lipid metabolism in AML. Through the modulation of key lipid synthesis-related genes, downregulating IRF1 led to a reduction in a wide range of lipids such as FA, SP, GP, and GL classes. Fatty acids, sphingolipids, and glycerophospholipids play important roles in cancer signaling and can promote the growth, metastasis, and drug resistance of certain tumor cells [60, 61].

However, this study has limitations. We only evaluated 11 patients in the present study. A larger number of patients should be included in future studies and more studies should be also conducted to confirm our findings. Furthermore, when employing CRISPR-Cas9 for knocking out the E1 region, our design incorporated a GFP fluorescent tag in the sgRNA plasmid without the inclusion of additional antibiotic resistance markers. This design constraint precluded the ability to efficiently screen for monoclonal populations. Additionally, it has been reported that MYC upregulation could promote the transcription of lipid metabolism genes and synergize with SREBP1 to regulate lipid synthesis in tumor cells [62]. However, whether the regulation of lipid metabolism by IRF1 is dependent on MYC remains to be further confirmed.

## Conclusion

Based on the above research, we identified IRF1 as a novel core transcription factor that forms AML-CRC with ELF1, ETV6, RUNX2, and MEF2D. The upregulation of IRF1 expression in AML is associated with poor prognosis, whereas the downregulation of IRF1 expression inhibits the proliferation of AML cells and promotes apoptosis. Furthermore, our findings indicate that IRF1 affects AML cell survival through the transcriptional regulation of key genes involved in lipid metabolism. Overall, our study provides new insights into the mechanisms of transcriptional regulation in AML and offers potential targets for AML treatment.

## Supplementary Information

Supplementary Material 1.

Supplementary Material 2.

Supplementary Material 3.

## Data Availability

Data is provided within the manuscript or supplementary information files. Additionally, RNA‑seq and CUT-Tag data have been submitted to the GEO database with accession numbers GSE268969 and GSE269001.

## Abbreviations

AML: Acute myeloid leukemia
CRC: Core transcriptional regulatory circuits
SEs: Super enhancers
TE: Typical enhancer
TF: Transcription factor
STR: Short tandem repeats
HE: Hematoxylin and eosin
IHC: Immunohistochemical
PCA: Principal component analysis
HSPC: Hematopoietic stem and progenitor cell
NC: Negative control
FA: Fatty acyls
SP: Sphingolipids
GP: Glycerophospholipids
GL: Glycerolipids
T-ALL: T-cell acute lymphoblastic leukemia
CLL: Chronic lymphocytic leukemia
