# Supplementary figures and images for: IRF1 is a core transcriptional regulatory circuitry member promoting AML progression by regulating lipid metabolism
Source: Exp Hematol Oncol. 2025 Mar 1;14:25. doi: 10.1186/s40164-025-00612-z (PMC11871635; doi:10.1186/s40164-025-00612-z)

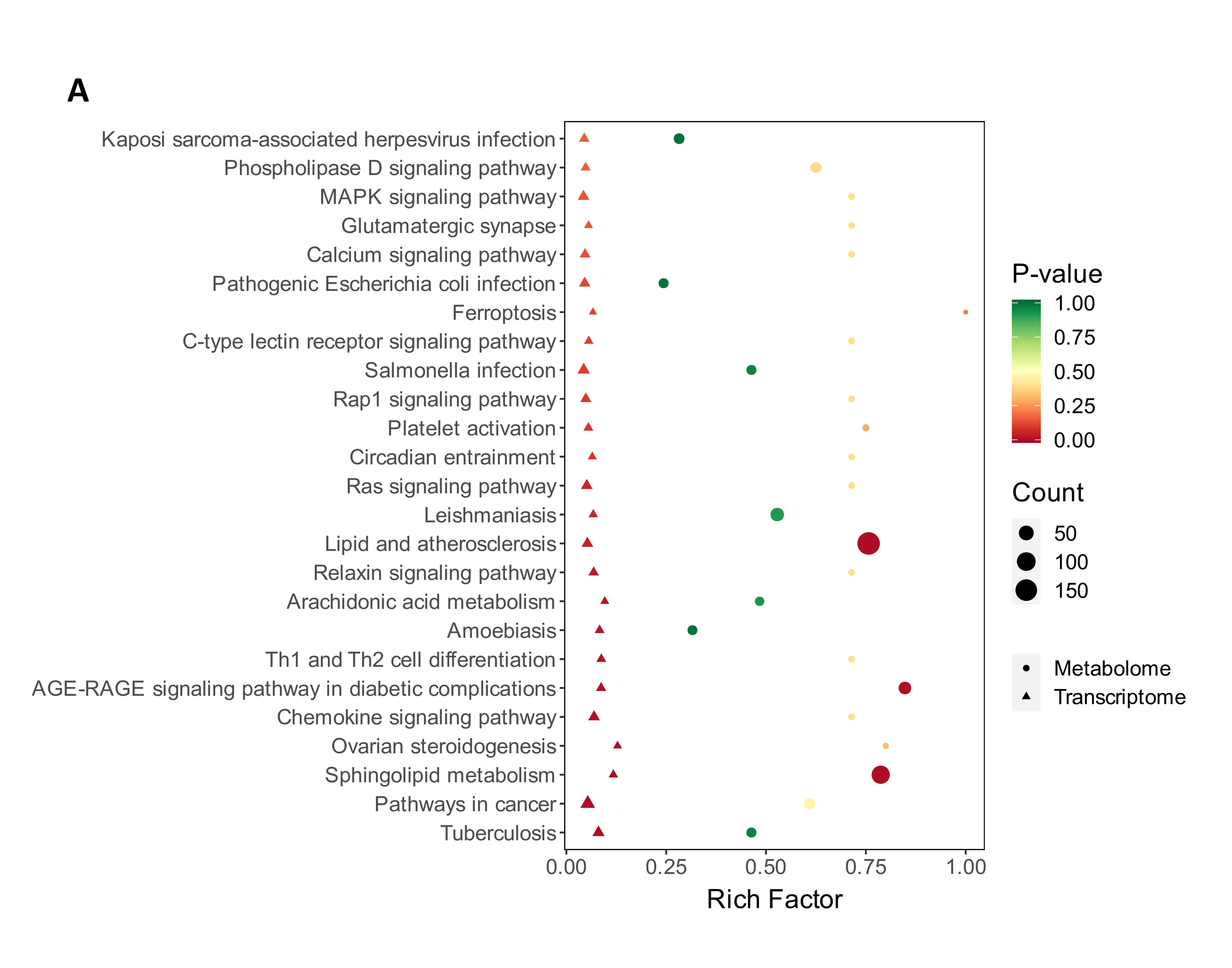

Supplement: Supplementary file 1 — Supplementary Material 1. [file 40164_2025_612_MOESM1_ESM.zip › Supplementary Figures/Supplementary Figure 14.jpg]

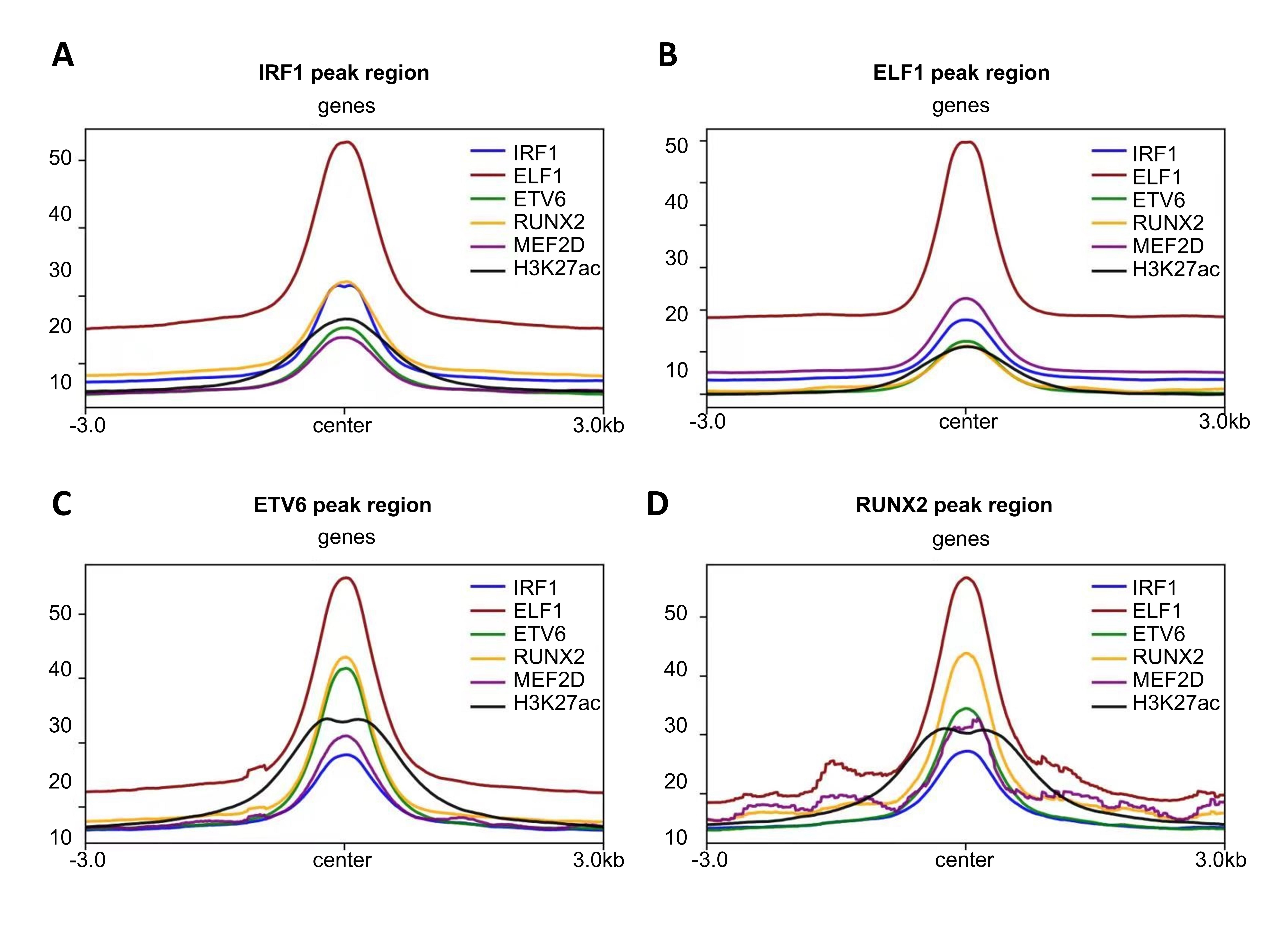

Supplement: Supplementary file 1 — Supplementary Material 1. [file 40164_2025_612_MOESM1_ESM.zip › Supplementary Figures/Supplementary Figure 3.jpg]

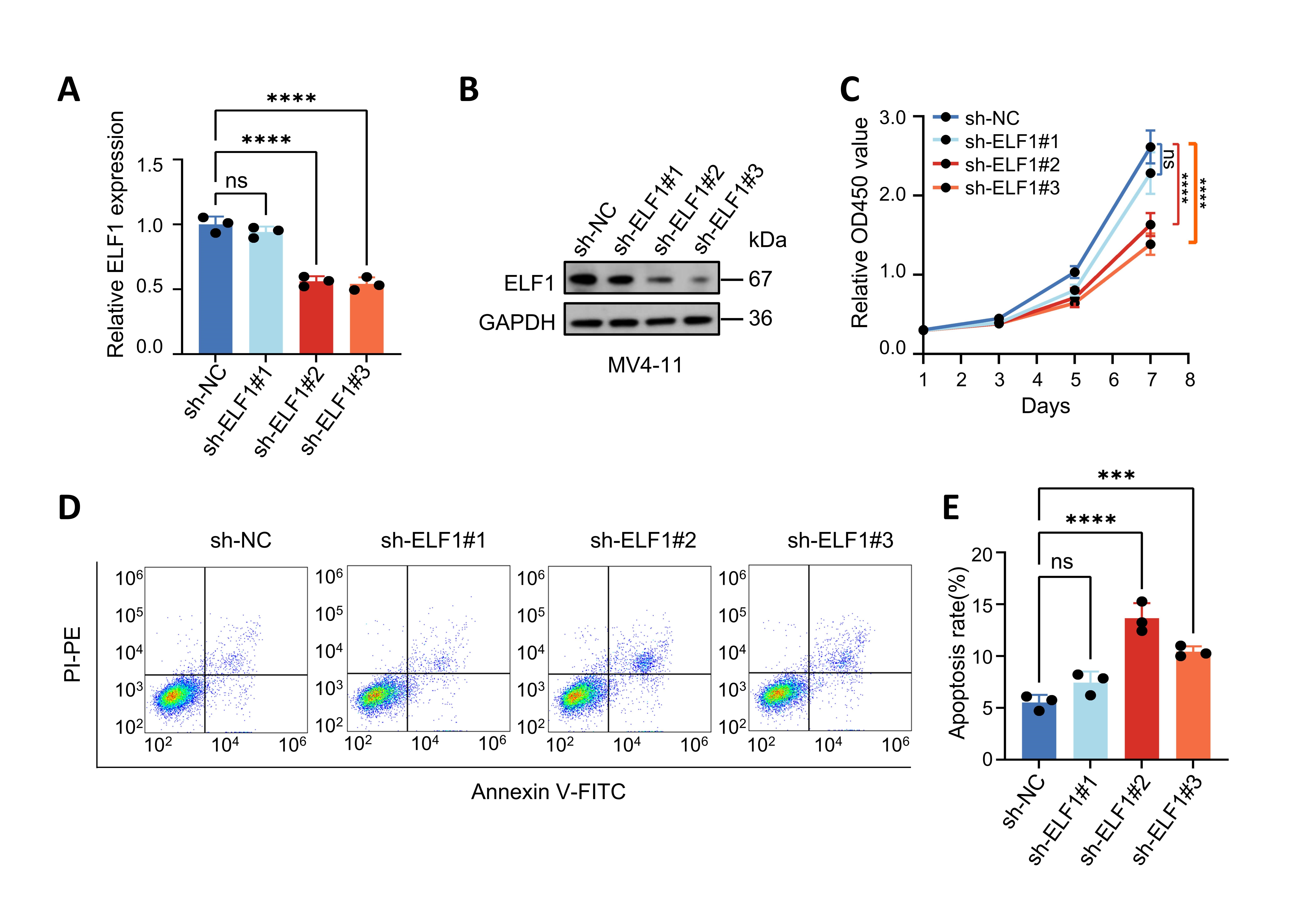

Supplement: Supplementary file 1 — Supplementary Material 1. [file 40164_2025_612_MOESM1_ESM.zip › Supplementary Figures/Supplementary Figure 5.jpg]
