# Supplementary material for: IRF1 is a core transcriptional regulatory circuitry member promoting AML progression by regulating lipid metabolism
Source: Exp Hematol Oncol. 2025 Mar 1;14:25. doi: 10.1186/s40164-025-00612-z (PMC11871635; doi:10.1186/s40164-025-00612-z)
Supplement: Supplementary file 3 — Supplementary Material 3. [file 40164_2025_612_MOESM3_ESM.docx]

Supporting Information

**IRF1 Is a Core Transcriptional Regulatory Circuitry Member Promoting AML Progression by Regulating Lipid Metabolism**

Fenli Zhang^1#^, Zhiheng Li^2#^, Fang Fang^2#^, Yixin Hu^3#^, Zhixu He^1^, Yanfang Tao^4^, Yizhen Li^3, 9^, Zimu Zhang^2^, Bi Zhou^5, 6^, Ying Yang^1^, Yumeng Wu^5, 7^, Yijun Wu^5^, Zhongling Wei^3^, Ailian Guo^3^, Ling Xu^5, 8^, Yongping Zhang^5^, Xiaolu Li^2^, Yan Li^5, 8^, Chunxia Yang^1^, Man Zhou^1^, Jian Pan^2^*, Shaoyan Hu^3^*, Xiaoyan Yang^1^*

**Supplementary Figure 1. The core of transcription factors in AML patients**

**(A)** Cluster analysis results of 11 AML samples, 3 AML cell lines, and 7 T-ALL cell lines based on the H3K27ac signals identified in each sample.

**(B)** CRC model based on H3K27ac ChIP-Seq data from 11 AML patients.

**(C-G)** The IGV plots of CUT&Tag and ChIP-Seq data show SE peaks (shaded regions) for ELF1, ETV6, RUNX2, and MEF2D near their respective genes.

**Supplementary Figure 2. Inflection plot ranking enhancer intensities**

**(A)** Inflection plots ranking all typical- and super-enhancers in MV4-11 and AML patients, and only AML CRC super-enhancers are displayed as examples.

**Supplementary Figure 3. Metagene analyses using MV4-11 CUT&Tag data.**

**(A)** Line plots showing the distribution of indicated CUT&Tag signals at IRF1 peak regions (centred at the summit of IRF1 peaks).

**(B)** Line plots showing the distribution of indicated CUT&Tag signals at ELF1 peak regions (centred at the summit of ELF1 peaks).

**(C)** Line plots showing the distribution of indicated CUT&Tag signals at ETV6 peak regions (centred at the summit of ETV6 peaks).

**(D)** Line plots showing the distribution of indicated CUT&Tag signals at RUNX2 peak regions (centred at the summit of RUNX2 peaks).

**Supplementary Figure 4. Expression and prognosis of five TFs in AML**

**(A)** Analysis of the GEPIA database reveals that IRF1, ELF1, ETV6, RUNX2, and MEF2D were overexpressed in AML.

**(B)** Kaplan-Meier curves are utilized to evaluate the AML patients’ survival according to the expression of IRF1, ELF1, ETV6, RUNX2, and MEF2D in the TCGA database.

**Supplementary Figure 5. Altered ELF1 expression affects the viability of AML cells**

**(A)** The knockdown levels of ELF1 in MV4-11 are verified by qRT-PCR.

**(B)** The knockdown levels of ELF1 in MV4-11 are verified by western blotting.

**(C)** CCK-8 assay detects the proliferation rate of MV4-11 cells after ELF1 knockdown.

**(D) (E)** Flow cytometry using Annexin V staining shows that knockdown of ELF1 increased the apoptotic rates of MV4-11 cell line.

**Supplementary Figure 6. Immune infiltration score of IRF1 in tumors**

**(A)** Stromal score of IRF1 in AML, BRCA, KIPAN and SKCM.

**(B)** Immune score of IRF1 in AML, BRCA, KIPAN and SKCM.

**(C)** ESTIMATE score of IRF1 in AML, BRCA, KIPAN and SKCM.

**Supplementary Figure 7. IRF1 exhibits elevated expression in AML**

**(A)** IRF1 is highly expressed in AML compared with other types of tumors.

**(B)** IRF1 mRNA expression levels in AML cell lines.

**(C)** The knockdown levels of IRF1 in AML cells are verified by qRT-PCR.

**(D)** The expression of IRF1 before and after virus infection is detected by western blotting.

**(E)** White slice shows that IRF1 knockdown significantly inhibited AML cell proliferation and promoted cell apoptosis.

**(F)** Downregulation of IRF1 causes a decrease in cyclin expression.

**Supplementary Figure 8. Knockdown of IRF1 results in attenuated proliferation of AML cells**

**(A)** Western blot verification of the knockdown efficiency of IRF1 in AML cells.

**(B)** CCK8 detection of cell proliferation in AML cells transfected with sh-NC or sh-IRF1 lentivirus.

**(C)** Colony formation assay of sh-NC or sh-IRF1 lentivirus transfected MV4-11 and Kasumi-1 cells.

**(D)** Cell cycle detection by flow cytometry after sh-NC or sh-IRF1 transfection of MV4-11 and Kasumi-1 cells.

**Supplementary Figure 9. Down-regulation of IRF1 promotes AML cell apoptosis**

**(A)** Statistical graph depicting the cell cycle distribution in AML cells transfected with sh-NC or sh-IRF1 lentivirus.

**(B-C)** Flow cytometry testing of apoptosis in AML cells transfected with sh-NC or sh-IRF1 lentivirus.

**Supplementary Figure 10. Downregulation of IRF1 inhibits AML progression in vivo**

**(A)** Bioluminescence imaging on days 15, 20, 25, 30 and 35 in the IRF1 knockdown group and control group injected Kasumi-1.

**(B-C)** Bioluminescence imaging and statistical analysis of liver, spleen, and bone in IRF1 knockdown group and control group injected Kasumi-1.

The percentages of CD45-positive cells in the liver, spleen and bone marrow of IRF1 knockdown mice are significantly decreased.

**(D-E)** Histograms show the bioluminescence signal values for both groups of mice at different time points.

**(F-G)** The picture of the two groups of mice liver and spleen.

**(H)** Comparison of liver and spleen weights between the two groups of mice.

**(I)** Survival curves of mice in both groups injected Kasumi-1(n=5).

**Supplementary Figure 11. Flow cytometry and HE staining were utilized to evaluate the infiltration of tumor cells in mice.**

**(A-D)** Flow cytometry was utilized to detect the infiltration of human CD45-positive cells in the liver, spleen, and femur of mice.

**(E)** HE staining was conducted to evaluate the infiltration of tumor cells in the liver, spleen, and femur of mice (tumor cells specifically indicated by yellow arrows).

**Supplementary Figure 12.** **The inhibitors of MYC and lipid metabolism suppress the growth of AML cells**

**(A**) The lipogenesis inhibitors Fatostatin, A939572, and Orlistat exhibit inhibitory effects on the growth of AML cells.

**(B)** The MYC inhibitor 10058F4 exhibits inhibitory effects on the growth of AML cells.

**(C)** The IC50 values for the four inhibitors.

**(D-E)** ZIP Synergy Scores for Combinations of c-MYC Inhibitor 10058F4 with Lipogenesis Inhibitors Fatostatin, A939572, and Orlistat.

**Supplementary Figure 13.** **Knockdown of IRF1 reduces the synthesis of multiple lipid molecules**

**(A**) PCA analysis of lipidomics samples.

**(B)** Downregulation of IRF1 results in decreased synthesis of various lipid molecules (one example per subtype listed).

**(C)** KEGG enrichment analysis of lipidomic results.

**Supplementary Figure 14. KEGG pathway enrichment analysis**

**(A)** KEGG enrichment analysis pathway map by jointly analyzing RNA sequencing and lipidomics data. The bubble plot represents KEGG pathways that were jointly enriched in both the transcriptomic and metabolomic data. This bubble plot is a five-dimensional visualization, where the x-axis and y-axis represent the enrichment factor (Diff/Background) and the KEGG pathway name, respectively. For KEGG pathways with more than 25 shared hits between the transcriptome and metabolome, the top 25 pathways by P-value are shown, with transcriptomic data given priority when there is overlap.

**Supplementary Table 1.** The GEO accession numbers for the data used in this study.

**Supplementary Table 2.** Details of shRNA sequence information utilized in this study.

The CRC genes of 11 AML patients are calculated according to the methods in the literature.

**Supplementary Table 3.** Details of all PCR primers utilized in this study.

**Supplementary Table 4.** Information of all antibodies utilized in this study.

**Supplementary Table 5.** The sequences of Ctrl, E1 and E2.

**Supplementary Table 6.** The sequences of Ctrl-sgRNA, E1-sgRNA.

**Supplementary Table 7.** The peaks identified by CUT&Tag analysis of IRF1 in MV4-11.

**Supplementary Table 8.** The peaks identified by CUT&Tag analysis of ELF1 in MV4-11.

**Supplementary Table 9.** The peaks identified by CUT&Tag analysis of ETV6 in MV4-11.

**Supplementary Table 10.** The peaks identified by CUT&Tag analysis of RUNX2 in MV4-11.

**Supplementary Table 11.** The peaks identified by CUT&Tag analysis of MEF2D in MV4-11.

**Supplementary Table 12.** The peaks identified by CUT&Tag analysis of IRF1 in Kasumi-1.

**Supplementary Table 13.** The peaks identified by CUT&Tag analysis of ELF1 in Kasumi-1.

**Supplementary Table 14.** The peaks identified by CUT&Tag analysis of ETV6 in Kasumi-1.

**Supplementary Table 15.** The peaks identified by CUT&Tag analysis of RUNX2 in Kasumi-1.

**Supplementary Table 16.** The peaks identified by CUT&Tag analysis of MEF2D in Kasumi-1.

**Supplementary Table 17.** Principal Component Analysis (PCA) clustering results of lipidomics samples.

**Supplementary Table 18.** Information on all lipid molecules detected by lipidomics.

**Supplementary Table 19.** KEGG pathway analysis of lipidomic results.

**Supplementary Table 20.** Clinical information of 11 AML patients.

**Supplementary Table 21.** 11 AML patients CRC genes predicted by the dbCoRC tool.

**Supplementary Table 22.** Differentially expressed genes detected by RNA-Seq in MV4-11 cells following IRF1 knockdown.

**Supplementary Table 23.** GSEA analysis results following IRF1 knockdown in MV4-11 cells.
